# Use of a random effects meta‐analysis in the design and analysis of a new clinical trial

**DOI:** 10.1002/sim.7948

**Published:** 2018-09-06

**Authors:** Hayley E. Jones, A. E. Ades, Alex J. Sutton, Nicky J. Welton

**Affiliations:** ^1^ Population Health Sciences, Bristol Medical School University of Bristol Bristol UK; ^2^ Department of Health Sciences University of Leicester Leicester UK

**Keywords:** Bayesian analysis, expected power calculations, heterogeneity, prior distributions, sample size calculations

## Abstract

In designing a randomized controlled trial, it has been argued that trialists should consider existing evidence about the likely intervention effect. One approach is to form a prior distribution for the intervention effect based on a meta‐analysis of previous studies and then power the trial on its ability to affect the posterior distribution in a Bayesian analysis. Alternatively, methods have been proposed to calculate the power of the trial to influence the “pooled” estimate in an updated meta‐analysis. These two approaches can give very different results if the existing evidence is heterogeneous, summarised using a random effects meta‐analysis. We argue that the random effects mean will rarely represent the trialist's target parameter, and so, it will rarely be appropriate to power a trial based on its impact upon the random effects mean. Furthermore, the random effects mean will not generally provide an appropriate prior distribution. More appropriate alternatives include the predictive distribution and shrinkage estimate for the most similar study. Consideration of the impact of the trial on the entire random effects distribution might sometimes be appropriate. We describe how beliefs about likely sources of heterogeneity have implications for how the previous evidence should be used and can have a profound impact on the expected power of the new trial. We conclude that the likely causes of heterogeneity among existing studies need careful consideration. In the absence of explanations for heterogeneity, we suggest using the predictive distribution from the meta‐analysis as the basis for a prior distribution for the intervention effect.

## 1 INTRODUCTION

Applications for funding for a new clinical trial increasingly require reference to or performance of a systematic review of the literature.^1^ Chalmers and Lau^2^ argued in 1996 that “meta‐analyses of the pertinent published trials of the same therapy should always be undertaken before the start of a new trial, and the results examined to help determine the design of a new trial or determine if a trial should be undertaken at all.” Some epidemiology journals now require consideration of how the results of a new study update the evidence base.^3^ A recent survey of trialists shows that many think they “should” be using evidence synthesis more than they do at present to inform both the design and analysis of a new trial.^4^

Most trials recently funded by the UK National Institute for Health Research Health Technology Assessment referenced a systematic review in their application,^5^, ^6^ suggesting increasing awareness of previous evidence.^7^, ^8^ Common uses of systematic reviews to inform trial design include justification of the intervention comparison, outcome definition, or duration of follow up.^4^, ^5^, ^6^ Occasionally, meta‐analysis has been used to inform the size of the intervention effect the trial is powered to detect and hence sample size calculations.^6^, ^9^, ^10^ None of these reviews mention explicit use of prior evidence on the likely intervention effect through Bayesian methods. However, existing evidence provides valuable information on the likely values that the treatment effect in a new trial might take. Furthermore, a new study will add to the evidence base, and the results should be interpreted accordingly.

A meta‐analysis of previous trials can be used to formulate a prior distribution for the intervention effect in a new trial.^11^ This prior distribution might be incorporated at the analysis stage and/or could be used in various ways in trial design. For example, an alternative to calculating the power to detect some prespecified intervention effect is to calculate the average or expected power across a prior distribution.^12^, ^13^, ^14^, ^15^ The “assurance” or “expected power” of a trial quantifies, for each sample size, the probability under the prior distribution of the new trial leading to a rejection of the null hypothesis.^16^ Expected power can be calculated even if the prior distribution will not be incorporated at the analysis stage: this has been called a “hybrid classical‐Bayesian” approach.^12^, ^13^, ^14^, ^16^ Alternatively, we can calculate the expected power of the new trial to impact upon the posterior distribution in a Bayesian analysis, which combines the prior information with the results from the trial.^12^ The latter is related to “pre‐posterior” analysis, a broader “fully” Bayesian approach, in which a loss function is averaged across a prior distribution.^17^, ^18^

It has also been proposed that one might calculate the power of a new trial to impact upon the pooled estimate from an updated meta‐analysis.^19^, ^20^, ^21^ Roloff et al^20^ provide formulae to estimate the power conditional on some prespecified true intervention effect, whereas Sutton et al^19^, ^21^ describe an approach to calculate the expected power to impact upon the meta‐analytic summary, averaged across a prior distribution.

Meta‐analysis models depend on the degree of heterogeneity in treatment effects between studies. Between‐study heterogeneity is common and is usually accommodated by use of a random effects model. This model is usually summarized with the estimated random effects mean. However, this focus has been questioned, and other summaries are available, including the predictive distribution and shrinkage estimates.^22^, ^23^, ^24^, ^25^ The random effects mean is an estimate of the average intervention effect across the set of trials in the meta‐analysis but might not represent any one trial population. Crucially, it might not represent the population of interest to the trialist.

In this paper, we take the perspective of a trialist planning a new randomized trial of an intervention versus control. We consider the situation where the trialist wants to utilize relevant information from a meta‐analysis of previous studies by powering the trial based on its ability either to (i) affect the posterior distribution in a Bayesian analysis of the trial data incorporating prior information from the meta‐analysis or (ii) impact upon the estimated mean in an updated random effects meta‐analysis. We describe in Section 2 how, in the presence of heterogeneity, these two approaches implicitly make different assumptions about the relationship between the new trial data and the trialist's target parameter (ie, the parameter that he or she aims to make inference on, defined by the patient population, intervention, comparator, and primary outcome; PICO). In Section 3, we discuss various possible interpretations of heterogeneity in the meta‐analysis and how these should impact upon the choice of approach *and* on the most appropriate prior distribution for a Bayesian analysis. In doing so, we draw on related arguments made about the choice of treatment effect estimate to incorporate into a health economic decision model.^26^, ^27^, ^28^, ^29^, ^30^ We demonstrate the large impact that the trialist's decisions on these matters can have in practice, using Bayesian expected power calculations in a hypothesis testing framework^12^, ^19^ as a simple example.

## 2 HOW DO THE HISTORIC DATA AND THE NEW DATA RELATE TO THE TRIALIST'S TARGET PARAMETER?

We assume that the trialist has a specific target parameter to estimate. In this section, we consider the relationship between this target parameter, the historic data, and the data from the new trial. We consider fixed effect and random effects meta‐analysis in turn.

For ease of exposition, we present formulae based on normal approximations whenever possible. Extensions to other likelihoods (eg, binomial likelihood for binary outcomes) are straightforward within a Bayesian statistical framework using Markov chain Monte Carlo simulation.^19^, ^31^

### 2.1 Fixed effect meta‐analysis

First, consider the case where the evidence from the previous trials was homogeneous, summarised with a fixed effect meta‐analysis model. This model assumes that the true effect of the intervention relative to a comparator, δ, is identical across all included studies, with all observed variability in effect estimates being due to sampling variation

$$y_{i}∼\text{Normal}\left(\delta ,{\mathrm{se}}_{i}^{2}\right),$$

where *y*_*i*_ is the estimated intervention effect (eg, log odds ratio) in study *i = *1*, …,m*, and ${\mathrm{se}}_{i}^{2}$ is an estimate of its variance (assumed known).

Even if there is no heterogeneity in the meta‐analysis, the trialist needs to consider carefully whether the target of inference in the new trial is the same parameter, δ. This might not be the case, for example, if all previous trials were in a different population or suspected to be at high risk of bias. However, if the trialist is indeed comfortable that the new trial will be homogeneous with the previous trials, then either of the following analyses might be planned:

- Basing an informative prior distribution for δ on the current summary estimate from the fixed effect meta‐analysis. Performing a Bayesian analysis of the new trial data, incorporating this prior distribution.
- Updating the fixed effect meta‐analysis to incorporate the new trial data.

These two analyses are equivalent. As such, it is equivalent to power the new trial based on its ability to impact upon either of these two analyses.

### 2.2 Random effects meta‐analysis

In practice, some degree of heterogeneity should be expected in many meta‐analyses.^32^ This can be accommodated with a random effects model, in which the intervention effects across different studies are assumed to be drawn from a common normal distribution rather than being identical

$$\begin{array}{l} y_{i}∼\text{Normal}\left(\delta_{i},{\mathrm{se}}_{i}^{2}\right)\\ \delta_{i}∼\text{Normal}\left(\mu ,\tau^{2}\right), \end{array}$$

where *μ* and *τ* denote the mean and standard deviation of the distribution of true intervention effects across studies. In published meta‐analyses, attention is generally focused primarily on the estimate of the mean *μ*.^33^ It has, however, been argued for many years that less emphasis should be placed on this estimate*,* which does not communicate the extent of variability between study results.^22^, ^23^, ^24^ The greater the amount of heterogeneity, the less meaningful is the random‐effects mean.

**Figure 1:**
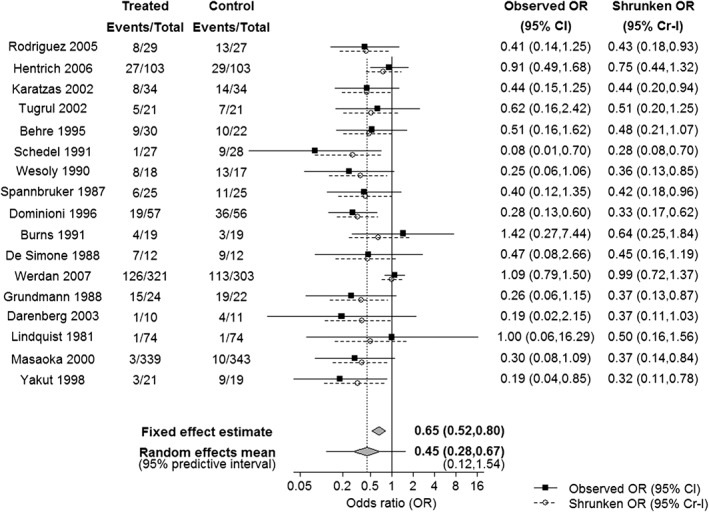
Forest plot showing results from a meta‐analysis of trials investigating the effect of intravenous immunoglobulin for severe sepsis and sepsis shock on all‐cause mortality, relative to standard care. Results are from Bayesian fixed and random effects meta‐analyses fitted in WinBUGS, assuming binomial likelihoods. The Bayesian random effects meta‐analysis accounts for uncertainty in the estimation of the between‐studies standard deviation τ, which is estimated as 0.54 (95% credible interval 0.26 to 1.02), ie, there is strong evidence of heterogeneity in the meta‐analysis. The dashed vertical line indicates the random effects mean

For example, Figure 1 shows data from 17 studies investigating the effectiveness of intravenous immunoglobulin (IVIG) versus standard care for severe sepsis and sepsis shock,^29^, ^34^ in the management of adult patients in intensive care. The outcome is all‐cause mortality. A fixed effect meta‐analysis estimates the odds ratio (OR) in all trials to be 0.65 (95% credible interval, Cr‐I, 0.52 to 0.80). However, there is strong evidence of statistical heterogeneity, such that a random effects meta‐analysis would generally be considered more appropriate. The summary OR from a random effects meta‐analysis is 0.45 (95% Cr‐I 0.28 to 0.67), indicating strong evidence that IVIG reduces mortality *on average* across studies. The between‐study standard deviation on the log odds ratio scale δ is estimated as 0.54 (95% Cr‐I 0.26 to 1.02).

Despite the existing strong evidence for an intervention effect “on average”, as there is heterogeneity it is unclear what the effect in any individual trial might be. There are many reasons therefore why a new trial of IVIG versus standard care might be planned: for example, to quantify the intervention effect in a specific patient population, or if estimates from some or all previous studies were considered likely to be biased away from the null.

Let us denote the trialist's target parameter by θ. He or she needs to consider carefully the relationship between this parameter and the information from the meta‐analysis. We will consider this in depth in the next section. For now, assume that some prior distribution for θ has been formulated based on the meta‐analysis

(1) $$\theta ∼\text{Normal}\left(\theta_{0},{\sigma_{0}}^{2}\right).$$

We now distinguish between the situation where the trialist's analysis of interest is (i) a Bayesian analysis of the new trial data, incorporating this prior distribution, or (ii) an updated random effects meta‐analysis. Unlike in the fixed‐effect case (Section 2.1), these two analyses are not equivalent. As such, powering the trial based on its ability to impact upon each of these analyses can imply very different necessary sample sizes. To demonstrate this, we provide formulae for “expected power” calculations in each case.

To help determine which of the two analyses should be of most interest to the trialist, we consider the implications of the choice of analysis for the assumed relationship between the new trial data and the target parameter, θ.

- Bayesian analysis of the new trial, incorporating the prior information

The new trial will provide data, for example, log odds ratio, with approximate normal distribution $y_{\mathrm{new}}∼\text{Normal}\left(\theta ,{\mathrm{se}}_{\mathrm{new}}^{2}\right)$. As in any standard sample size or power calculation, we re‐express ${\mathrm{se}}_{\mathrm{new}}^{2}$ as σ^2^/n, where n is the number of patients in the new trial and σ is the standard deviation of patient‐level outcomes. Some value needs to be assumed for this, such as the median value of ${\mathrm{se}}_{i}\sqrt{n_{i}}$ across previous trials.

We can also re‐express the prior variance (Equation (1)) in this format, ie, σ_0_^2^ = σ^2^/n_0_. This allows the amount of information contained in the prior distribution to be quantified as equivalent to trial data on n_0_ patients.

A Bayesian analysis, incorporating the prior distribution, will produce the following posterior distribution for θ:

$$\theta \;|\;y_{\mathrm{new}}∼\text{Normal}\left(\frac{n_{0}\theta_{0}+{\mathrm{ny}}_{\mathrm{new}}}{n_{0}+n},\frac{\sigma^{2}}{n_{0}+n}\right).$$

Let θ < 0 indicate a benefit of the intervention relative to the control. Say that the new trial will test the hypothesis H_0_ : θ > θ^*^ versus H_1_ : θ < θ^*^, for some θ^*^ ≤ 0. For example, θ^*^ might be zero, or the minimal clinically important difference (MCID). The “Bayesian significance” can be defined as the posterior probability of the null hypothesis being less than some cut‐off, say α, ie, the null hypothesis will be rejected if Pr(θ > θ^*^| y) < α.^12^

The “expected power,” ie, the expected probability of a significant result, averaging across the prior distribution for θ , (expression (1)) is^12^ (see also our Appendix)

(2) $$1-\Phi \left(\sqrt{\frac{n_{0}}{n}}\left(z_{1-\alpha }+\frac{\left(\theta_{0}-\theta^{*}\right)\sqrt{n_{0}+n}}{\sigma }\right)\right),$$

where Φ denotes the distribution function of the standard normal distribution and z_1 − α_ is its 100 × (1 − α) percentile.

As n increases to infinity, the expected power tends to

(3) $$1-\Phi \left(\frac{\theta_{0}-\theta^{*}}{\sigma /\sqrt{n_{0}}}\right),$$

the prior probability that θ < θ^*^. Alternatively, as n_0_ tends to 0 (representing no prior information), expression (2) tends to 0.5, representing true clinical equipoise.

ii Updated random effects meta‐analysis

We now consider the situation where the trialist's main analysis of interest is an updated random effects meta‐analysis. Following approaches suggested in other works,^19^, ^20^, ^21^ he or she might then power the new trial based on its ability to impact upon the estimated random effects mean. It has been shown that doing so has some counter‐intuitive consequences.^19^, ^28^ In particular, if there is considerable heterogeneity in the meta‐analysis then a new trial, however large, has little ability to change the estimate of the random effects mean, and so might not be considered worthwhile.^19^, ^35^ Further, multiple smaller studies may be more “powerful” than one larger study.^19^, ^20^, ^28^, ^36^

An updated estimate of the random effects mean will not be the same as the result from a Bayesian analysis of the new trial data unless τ = 0, ie, there is no heterogeneity. In order for the two approaches to be equivalent, we would have to assume that the new trial data will not directly estimate the target parameter (θ): a rather strange concept in most scenarios. Powering the trial based on its ability to affect the random effects mean implicitly assumes that a new study of size n provides data y_new_ ∣ δ∼Normal(δ, σ^2^/n) where δ∼Normal(θ, τ^2^), or, equivalently, y_new_∼Normal(θ, (σ^2^/n) + τ^2^). That is, the variance of the assumed likelihood is essentially inflated by an additive factor of τ^2^. See the Appendix for more details. The ability of the new study to affect inference about θ is limited by this assumed additional variability.

In Section 3, we will discuss when, if ever, it might be appropriate for a trialist to power the trial based on its ability to affect the random effects mean. For now, let us assume that this is the analysis of interest. Sutton et al^19^, ^37^ describe a flexible simulation‐based approach to calculating the expected power of a new study to influence the random effects mean. In the Appendix, we derive a closed‐form approximate formula based on the same principles, for the special case where there is a normal likelihood and the between‐studies standard deviation τ is treated as known.

The expected probability of rejecting the null hypothesis that θ > some value θ^*^is then

(4) $$1-\Phi \left(\sqrt{\frac{\left(\sigma^{2}/n\right)+\tau^{2}}{\left(\sigma^{2}/n_{0}\right)}}z_{1-\alpha }+\left(\frac{\theta_{0}-\theta^{*}}{\left(\sigma /\sqrt{n_{0}}\right)}\right)\sqrt{1+\frac{\left(\sigma^{2}/n\right)+\tau^{2}}{\left(\sigma^{2}/n_{0}\right)}}\right).$$

As we would expect, this reduces to expression (2) if τ = 0. As n increases to infinity, the expected power tends to

(5) $$1-\Phi \left(\sqrt{\frac{\tau^{2}}{\left(\sigma^{2}/n_{0}\right)}}z_{1-\alpha }+\left(\frac{\theta_{0}-\theta^{*}}{\left(\sigma /\sqrt{n_{0}}\right)}\right)\sqrt{1+\frac{\tau^{2}}{\left(\sigma^{2}/n_{0}\right)}}\right),$$

which reduces with increasing τ and has a maximum of $1-\Phi \left(\frac{\theta_{0}-\theta^{*}}{\left(\sigma /\sqrt{n_{0}}\right)}\right)$ (= expression (3)) when τ = 0.

Demonstration of the impact of the choice of analysis.

The impact of the choice between analysis (i) and (ii) will be large in the presence of considerable heterogeneity in the meta‐analysis. For example, say that we were planning a new trial of IVIG versus standard care. Let us say, hypothetically, that the minimum clinical difference to change practice was θ^*^ =  log (0.6) =  −0.51. We note that this is probably an unrealistically large MCID given that the outcome is mortality, but we have selected a value within the 95% Cr‐I for the current estimate of the random effects mean for demonstration purposes.

Let us assume for now that we have based our prior distribution for the intervention effect on the current random effects mean: we will discuss other options in Section 3. We approximated this by a Normal(−0.81, σ^2^/415) distribution for the log odds ratio. Note that the amount of information contained in this prior distribution is equivalent to trial data on 415 patients. Say that we will reject the null hypothesis if the posterior probability is at least 95%, ie, α = 0.05 so that z_1 − α_= 1.65.

**Figure 2:**
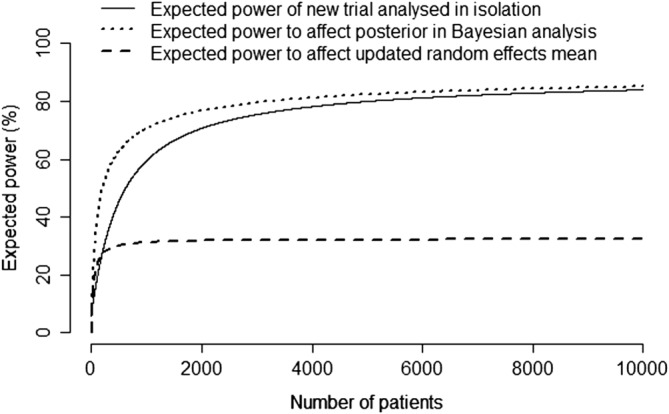
“Expected power” of a new study of intravenous immunoglobulin versus control, in testing the hypothesis H_0_ : OR > 0.6 versus H_1_ : OR < 0.6. We take α = 0.05 and assume σ = 4.47, estimated as $\text{median}\left({\mathrm{se}}_{i}\sqrt{n_{i}}\right)$ across trials in the meta‐analysis, where se_i_ is the standard error of the log odds ratio and n_i_ is the number of patients in trial i. We averaged across the following prior distribution for the target parameter: θ ∼ Normal(−0.81, σ^2^/415). The following expected power calculations are then shown: (i) hybrid classical‐Bayesian, in which the new trial is analyzed in isolation; (ii) power to affect the posterior distribution in a Bayesian analysis (expression (2)); (iii) power to impact upon the random effects mean (expression (4)). In applying expression (4), we assumed a fixed value of τ = 0.54, the estimate from the meta‐analysis

As a baseline case, on Figure 2 we show the expected power of the new trial based on the hybrid classical‐Bayesian approach, ie, if the new trial data will be analyzed in isolation, without incorporating any previous information from the meta‐analysis (see Spiegelhalter et al^12^ for formulae). We also show the expected power of the new trial to impact upon the posterior distribution in a Bayesian analysis (approach (i): expression (2)). Each of these has a maximum possible value of 91%, which is the prior probability of H_1_ (OR < 0.6) (expression (3)). The expected power is greater, especially at smaller sample sizes, if the prior information will be incorporated at the analysis stage.

If, instead, approach (ii) is taken, ie, the trial is powered on its ability to impact upon the updated random effects mean, the maximum expected power is only 32% (expression (4)). Given the heterogeneity in the meta‐analysis, a new trial has very little ability to impact upon the random effects mean so would almost certainly not be considered worthwhile. Further, as shown in Figure 2, the expected power is already close to its theoretical maximum for a sample size of 500 patients so that there is essentially no benefit of a larger sample size than this.

## 3 INTERPRETATIONS OF HETEROGENEITY: IMPLICATIONS FOR THE CHOICE OF ANALYSIS AND CHOICE OF PRIOR DISTRIBUTION

Although heterogeneity in a meta‐analysis can be notoriously difficult to explain, it is helpful to at least conceptualize the various possible causes to determine how a given meta‐analysis might be used most appropriately in trial design.

We distinguish between four potential sources of heterogeneity, building on an earlier categorisation of Ades et al^26^ and previous distinctions made between “true” and “artefactual” variation,^24^, ^38^ also termed “diversity” and “bias.”

### 3.1 Variation in the target parameter

Trials included in a meta‐analysis will tend to vary in some aspects of their protocols,^25^, ^39^ for example, recruiting from patient populations that differ in terms of baseline severity or demographic factors. These factors only translate into heterogeneity if they interact with the intervention effect. As each trial in the meta‐analysis will have been designed to estimate the intervention effect for a specific PICO, this means that the target parameter varies across trials.

The trialist's own target of inference, θ, will be the intervention effect relating to a specific patient population and protocol. Clearly, the new trial will be designed to estimate *θ* directly, such that an updated random effects meta‐analysis is not the analysis of interest to the trialist. However, a Bayesian analysis of the new trial data incorporating an informative prior distribution based on the meta‐analysis might be planned. The average intervention effect across the previous diverse set of studies (the random effects mean) is unlikely to be a good prediction of the effect in the new trial. As such, it will not generally be appropriate to base the prior distribution for θ on the existing random effects mean. We suggest that it will generally be much more appropriate to base the prior on one of the following.

1. *Predictive distribution*

Given estimates of *μ* and *τ* from the existing data, the predictive distribution for the intervention effect in a new study, drawn from the distribution of random effects, is

$$\delta_{\mathrm{new}}|\mu ,\tau ∼\text{Normal}\;\left(\mu ,\tau^{2}\right).$$

This can be summarized by an interval indicating the likely range in which the true effect in a future study might lie, given the heterogeneity in the meta‐analysis.^23^, ^24^ For example, for the IVIG meta‐analysis, the predicted OR in a new study is 0.45 but with a wide 95% predictive interval running from 0.12 to 1.54 (Figure 1). If the trialist thinks it likely that the intervention effect varies according to trial characteristics but the precise sources of this variation are unknown, we suggest this would be the most sensible basis of a prior for the intervention effect in a new trial. This avoids making the strong assumption that the effect in the new trial will be the same as the average.

2. *Posterior distribution/shrinkage estimate*

For estimated *μ* and *τ* from data *D* (defined as the set of data on all *m* studies), the posterior distribution of the intervention effect in study *i* is^23^

$$\delta_{i}|\mu ,\tau ,D\;∼\;N\left(\frac{\tau^{2}y_{i}+{\mathrm{se}}_{i}^{2}\mu }{\tau^{2}+{\mathrm{se}}_{i}^{2}},\frac{\tau^{2}{\mathrm{se}}_{i}^{2}}{\tau^{2}+{\mathrm{se}}_{i}^{2}}\right).$$

An estimate of the posterior mean for a particular study is known as the shrinkage estimate since each posterior intervention effect is shrunk toward the average *μ* compared with the observed effect. The posterior variance is also reduced relative to ${\mathrm{se}}_{i}^{2}$ by “borrowing strength” from the other studies. On Figure 1 we show shrinkage estimates with 95% credible intervals for each of the IVIG studies. For example, we see that the posterior estimate for the Werdan trial is an OR of 0.99 (95% Cr‐I 0.72 to 1.37). Thus, even after the shrinkage, there is no evidence of an intervention effect in that study.

If the target parameter is believed to vary across trials in the meta‐analysis, the shrinkage estimate from the trial with characteristics most similar to the new trial (ie, addressing the most similar PICO) might be an appropriate basis for a prior distribution.

3. *Meta‐regression*

Ideally, observed heterogeneity should be explained rather than simply accommodated within a random effects meta‐analysis.^22^, ^25^, ^40^, ^41^, ^42^ This might be possible to some extent using meta‐regression, in which the intervention effect is regressed on one or more recorded study characteristics.^43^ A random effects meta‐regression is usually used, which allows for some degree of residual, unexplained heterogeneity in the meta‐analysis. This takes the form

$$\delta_{i}∼\text{Normal}\left(\alpha +\beta C_{i},\tau_{R}^{2}\right),$$

where *C*_*i*_ is the recorded study characteristic and *τ*_*R*_ is the (residual) standard deviation of true effects around the regression line. Alternatively, the regression might be on a latent study characteristic such as the underlying control arm risk, taken as a measure of baseline severity.^44^ If some of the heterogeneity is explained in this way, it makes sense to tailor the prior distribution for the new trial to the most relevant point on the meta‐regression line.

For example, Welton et al found the duration of IVIG treatment to be associated with the estimated intervention effect.^29^ Say, for instance, that the duration of treatment in the new trial will be three days. A meta‐regression model estimates that, on average, studies with this duration of treatment produce OR = 0.30 (95% Cr‐I 0.16 to 0.54), an estimate further from the null than the random effects mean. However, there is residual heterogeneity around the meta‐regression line: *τ*_*R*_ is estimated to be 0.35 (95% Cr‐I 0.02 to 0.88). A 95% predictive interval for the intervention effect in a new study with this duration of treatment is 0.10 to 0.89, narrower than the full predictive interval above since some of the heterogeneity has been accounted for. In the presence of such residual heterogeneity, we would recommend taking the predictive distribution around the most relevant point of the meta‐regression as the prior.

### 3.2 Measurement error

If all trials in the meta‐analysis addressed precisely the same PICO or if there is no reason to believe that the intervention effect will interact with any such factors that varied, then the target parameter is the same across the set of trials. Heterogeneity could however result from errors (beyond the random error due to sampling variability) in individual trials' estimates of this target parameter. We first consider the case where these errors are random and have an expectation of zero. We will refer to this as measurement error. This might result, for example, from “standardizing” effect estimates by dividing by the sample standard deviation. In addition to random variation in the standard deviation as a sample statistic, variation between trials in population standard deviations can be expected,^45^, ^46^, ^47^ such that even infinitely large trials with identical mean differences would produce different estimates of the standardized mean difference. Differences between test instruments in responsiveness would contribute further measurement error.^48^

If all heterogeneity in the meta‐analysis was due to random errors with a Normal(0, *τ*^2^) distribution, then the random effects mean would have a valid interpretation as the single true intervention effect, in the same way as the pooled result from a fixed effect meta‐analysis. The current random effects mean would then be the most appropriate basis for a prior distribution for this target parameter. However, if the measurement error cannot be avoided, then the new trial is expected to estimate *δ*∼Normal(*θ*, *τ*^2^) rather than estimating *θ* directly.

In this special (but somewhat unrealistic) situation, the updated random effects mean would correctly estimate the trialist's target parameter. The trialist could therefore appropriately power the new trial based on its ability to impact upon the random effects mean, using one of the approaches described by Sutton et al^19^, ^21^ (or our closed form approximation of this: expression (4)) or Roloff et al.^20^

### 3.3 Bias

Consider again the case where all trials in the meta‐analysis have tried to estimate the same target parameter but have done so with error. Often, such errors are systematic, ie, with a non‐zero expectation, such that estimates of the intervention effect are *biased*. This could result from methodological flaws in trial conduct such as inadequate blinding, for example.

The trialist aims to estimate the same target parameter as the previous trials. However, unless the potential flaw will also be present in the new trial, then the new trial is expected to estimate this directly, not with the additional random error that the heterogeneity parameter τ represents. As such, clearly, the updated random effects mean is not the target parameter of interest. Further, the current estimate of the random effects mean is a biased estimate of the target parameter and therefore a poor basis for a prior distribution. The prior distribution might instead be based on analysis of a single study or subset of studies assessed to be at low risk of bias. Alternatively, the prior distribution could be based on a bias‐adjusted meta‐analysis.

Say that a recorded study characteristic represents presence or absence of a marker of risk of bias (for example, inadequate blinding, method of randomization, or allocation concealment), such that there are *m*_*L*_ bias‐free studies and *m*_*H*_ studies at risk of bias. Denoting the observed intervention effects from these studies by $y_{i}^{L}$ and $y_{i}^{H}$, respectively, Welton et al^49^ proposed bias‐adjusted meta‐analysis models, including

$$\begin{array}{ll} y_{i}^{L}∼\text{Normal}\left(\delta ,s_{i}^{2}\right),\; & i=1,\text{…},m_{L}\\ y_{i}^{H}∼\text{Normal}\left(\delta +\beta_{i},s_{i}^{2}\right),\; & i=\left(m_{L}+1\right),\text{…},\left(m_{L}+m_{H}\right)\\ \;\beta_{i}∼\text{Normal}\left(b,\kappa^{2}\right),\; & i=\left(m_{L}+1\right),\text{…},\left(m_{L}+m_{H}\right).\; \end{array}$$

External evidence can be used to derive informative prior distributions for the average bias in the meta‐analysis, *b*, and the between‐study standard deviation in bias, *κ*,^49^ aiding estimation of the true intervention effect, δ. Alternatively, rather than assuming that the biases are randomly distributed across studies as above, the magnitude of these can be prespecified (with uncertainty) by experts, based on in‐depth consideration of the study characteristics.^50^ In the same way as the random effects meta‐regression model allows for some residual heterogeneity not explained by the recorded study characteristic, the bias model above can be extended to allow for additional unexplained heterogeneity.^49^, ^50^

For example, in the IVIG meta‐analysis, several risk of bias indicators such as inadequate blinding or allocation concealment were found to be important predictors of the intervention effect estimate. See the original systematic review for risk of bias tables.^34^ In an analysis allowing for bias in studies that were inadequately blinded, using informative priors for *b* and *κ* based on the BRANDO meta‐epidemiological study,^51^ we estimated the average intervention effect across adequately blinded studies to be OR = 0.50 (95% Cr‐I 0.31 to 0.78), slightly closer to the null than the random effects mean. Taking into account the considerable residual heterogeneity in the meta‐analysis after accounting for these potential biases, the predictive interval for a new adequately blinded study remains wide, from 0.15 to 1.66. Again, in the presence of residual heterogeneity, we would recommend using the predictive distribution around the relevant point as a prior.

### 3.4 Distribution of effects

Even if all studies have identical case mix and an identical protocol, with some interventions variation due to random deviations from this protocol or varying unknown skill or training levels of staff involved in administering the intervention is virtually inevitable. We refer to this as a “distribution of effects”: although the intervention effect varies, the heterogeneity is random, cannot be explained by covariates, and would be expected to continue if the intervention was rolled out on a large scale.

A meta‐analysis in which studies differed only in this way would be comparable to a multicenter trial: in such trials, between‐center differences are often observed, showing just how difficult it can be to replicate the same protocol in multiple centers. Variation due to random staff effects is conceivable particularly with complex interventions, for example, for surgical or psychological interventions.^52^, ^53^

Consider the case where all heterogeneity in the meta‐analysis is assumed to be due to such an inherent distribution of intervention effects, such that even with an identical patient population and protocol all of the heterogeneity is considered inevitable in clinical practice. In this hypothetical scenario, the effect in the new study would be of no more, or less, interest than that in any previous study. The random effects mean would be interpretable as the true average effect of the intervention. Similar to the “measurement error” situation, the parameter estimated in the new trial is therefore not the target parameter of interest. Instead, we can envisage that the analysis of interest is an updated random effects meta‐analysis. However, in contrast to the “measurement error” situation, the entire distribution of effects in the updated meta‐analysis is of interest, ie, the between studies variance parameter as well as the random effects mean. Other measures representing the entire distribution of effects are also likely to be of interest, for example, the proportion of studies or centres showing a clinically meaningful effect.

### 3.5 Demonstration of the impact of choice of prior distribution informed by the meta‐analysis

From the preceding subsections, we conclude that, in most situations, an updated random effects meta‐analysis is not the analysis of interest to the trialist. A Bayesian analysis of the new trial data incorporating prior information from the meta‐analysis would generally be more appropriate. We have also highlighted that the random effects mean is unlikely to be the most appropriate basis for a prior distribution, and we have discussed several more suitable alternatives.

**Figure 3:**
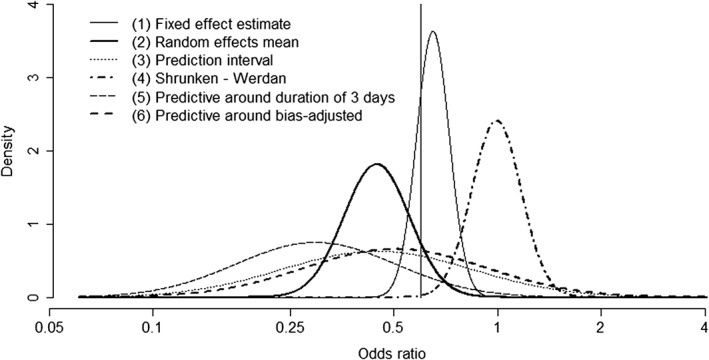
Six possible prior distributions for the intervention effect in a new trial of intravenous immunoglobulin versus standard care. The vertical line is drawn at OR = 0.6. Consider that in the new trial we planned to test H_0_ : OR > 0.6 versus H_1_ : OR < 0.6. The area under each curve to the left of the vertical line then indicates the prior probability of H_1_. All six prior distributions shown are based on normal approximations to results from Bayesian analyses of the previous data (fixed and random effects meta‐analysis, meta‐regression with duration as a covariate, and an analysis adjusting for inadequate blinding in some previous studies)

Table 1 summarizes the characteristics of the various prior distributions for a potential new trial of IVIG versus standard care that we have discussed. We include the shrinkage estimate for the Werdan trial since this was the most recent trial and was assessed to be at low risk of bias for all domains, so could realistically be considered the most relevant. This is not, of course, an exhaustive list of options. In each case, we approximated the point estimate and 95% prior interval given above by a normal distribution on the log odds ratio scale to apply expression (2). For completeness, we also include a prior distribution based on the fixed effect summary estimate. These six prior densities are displayed in Figure 3.

**Table 1:** Characteristics of six possible prior distributions for the intervention effect in a new trial of intravenous immunoglobulin versus standard care. All six prior distributions shown are based on normal approximations to results from Bayesian analyses of the previous data. The implicit sample size n_0_ is defined as σ^2^ divided by the variance of the prior distribution, where σ = 4.47, estimated as $\text{median}\left({\mathrm{se}}_{i}\sqrt{n_{i}}\right)$ across trials in the meta‐analysis

Source of Prior	Prior Estimate for	Prior Mean	Implicit	Prior
	OR in New Trial	log(OR)	Sample	Probability
	(95%prior interval)	(***θ***_0_)	size (n_0_)	OR < 0.6
(1) Fixed effect meta‐analysis estimate	0.65 (0.53, 0.80)	−0.43	1661	0.24
(2) Random effects meta‐analysis mean	0.45 (0.28, 0.67)	−0.81	415	0.91
(3) Predictive distribution	0.45 (0.12, 1.54)	−0.81	50	0.68
(4) Posterior / shrunken estimate from the Werdan study	0.99 (0.72, 1.37)	−0.01	731	0.00
(5) Predictive distribution around duration of three days	0.30 (0.10, 0.89)	−1.22	71	0.91
(6) Predictive distribution around bias‐adjusted meta‐analysis estimate	0.50 (0.15, 1.66)	−0.68	54	0.61

**Figure 4:**
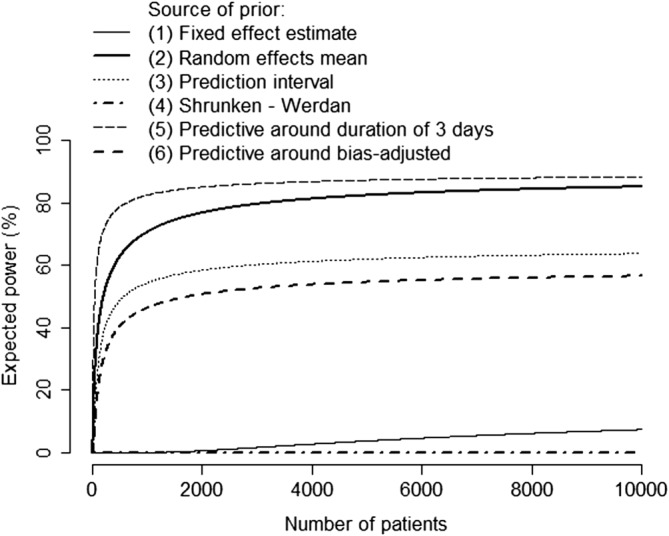
“Expected power” of a new study of intravenous immunoglobulin versus control, in testing the hypothesis H_0_ : OR > 0.6 versus H_1_ : OR < 0.6. We assume here that the target of inference is the intervention effect in the new trial not the random effects mean (ie, we apply expression 2 not 4). The plot demonstrates the impact of choice of prior distribution. The six prior distributions are described in Table 1 and Figure 3. We take α = 0.05 and assume σ = 4.47

Expected power calculations will of course be highly sensitive to the choice of prior distribution if the possible priors vary considerably (as is the case here). Figure 4 shows the results of applying expression (2) under each of these six prior distributions, assuming as before that our hypothesis of interest is *H*_0_ : *OR* > 0.6 versus *H*_1_ : *OR* < 0.6. We see, for example, that the expected power of a very large trial (10,000 patients) ranges from 0% to 88% across the six prior distributions. The expected power is much lower when the predictive distribution is used compared with the prior based on the random effects mean. This is despite these two prior distributions having the same mean, and arises because the predictive distribution has a much lower implicit sample size (equivalently, is less precise) such that its potential impact is limited. The prior distribution based on the Werdan study indicates that there is zero probability of the alternative hypothesis H_1_ (Table 1), such that the expected power of any new trial to reject H_0_ is zero. Although the implicit sample size of the predictive distribution around the meta‐regression line is low, the expected power is seen to be higher than in the other cases described because of the more optimistic prior mean.

## 4 DISCUSSION

In using a random effects meta‐analysis in the design or analysis of a new trial, trialists must remember that the random effects model estimates a *set* of intervention effects, varying across studies.^23^, ^24^, ^25^ Systematic reviews and meta‐analyses provide not just a single estimated intervention effect but a lens through which all available evidence can be viewed.^54^ The random effects mean is only an estimate of the average intervention effect across a set of studies, which should not generally be expected to be applicable to a new trial.

The trialist needs to decide in advance whether (i) the new trial will directly estimate the target parameter or (ii) the new data will be no more or less relevant than the data from any of the previous trials in the meta‐analysis, such that the main analysis of interest is an updated random effects meta‐analysis. While we envisage that the former will usually be the case, we have described two special situations where the latter might be true. First, the updated estimate of the random effects mean would be the most appropriate estimate of the trialist's target parameter if all heterogeneity in the meta‐analysis was believed to be due to “measurement error” centered on 0, which seems generally unlikely in practice. Second, an updated random effects meta‐analysis could also be the main focus of interest if there is in reality a distribution of effects that would be expected to always persist in clinical practice. However, this distribution of effects depends on both the mean and between studies variance parameters, and a new trial will provide information on both of these. Further work is needed to extend the ideas of expected power to the situation where a distribution of effects is of interest.

If the updated random effects meta‐analysis is indeed the main focus of interest, the seemingly anomalous result that multiple small new studies could be more powerful than one large study^19^, ^20^ makes sense: this would allow us to better estimate the true effect (after removing the “measurement error” expected in the new study) or to learn more about the entire distribution of effects expected in practice.

If, instead, the new trial will directly estimate the target parameter of interest, a prior distribution for this can be formed based on the random effects meta‐analysis. However, basing the prior on the current estimate of the random effects mean will not generally be appropriate. More suitable alternatives might include the predictive distribution, the posterior distribution of the intervention effect from the most relevant previous study, the predictive distribution around a meta‐regression line or a bias‐adjusted meta‐analysis.

In reality, multiple sources of heterogeneity are likely to be present in a meta‐analysis. Although in theory a model can be formulated to properly account for any combination of sources of heterogeneity in a meta‐analysis; in practice, attempts to do so using aggregate data are limited by low power, false positive findings, confounding, or lack of reporting of relevant variables. Clinical or other substantive input is crucial. For the IVIG example, the best‐fitting model was found to be one in which both treatment duration (or an alternative study characteristic relating to the dosing regimen) and a risk of bias indicator were accounted for. However, an expert advisory group indicated that there was no clinical rationale for the importance of the dosing variables.^34^ As such, it was concluded that a model accounting only for bias but allowing also for residual heterogeneity was the most appropriate. More formal incorporation of expert opinion is also possible but risks criticisms of subjectivity.^55^ Ideally, individual participant data should be used to explore heterogeneity in more depth.^51^

If some or all heterogeneity in the meta‐analysis remains unexplained, we would suggest the default approach might be to assume that the new trial data will directly estimate the target parameter and to take the predictive distribution (possibly around a meta‐regression line or bias‐adjusted analysis) as a prior. This allows for uncertainty that is ignored if basing a prior distribution on the average across a set of previous studies and assumes that the new trial is more relevant to the specific hypothesis of interest than any given trial in the meta‐analysis. A difficulty arises if there are very few trials in the meta‐analysis, such that the amount of heterogeneity is not well estimated, or if results from only a single previous trial are available, such that the true amount of heterogeneity across studies is completely unknown. A potential solution to this problem would be to use empirically based prior distributions for *τ* 32 when formulating an appropriate predictive distribution.

In this paper, we have taken the perspective of someone designing a new clinical trial. We recognize that others might later use the trial data for secondary analyses and these analysts may have a different focus: specifically, they might be more interested in an updated meta‐analysis. However, from the perspective of the people actually responsible for designing the trial, we have argued that an updated meta‐analysis will not usually be of primary interest.

The issues raised in this paper are also relevant to sequential methods for meta‐analysis such as “trial sequential analysis,” which involve repeatedly assessing whether a meta‐analysis is “conclusive” following the completion of each new trial.^36^, ^49^, ^50^, ^56^ These methods are similarly based on the questionable key assumption that the random effects mean is the target parameter of interest.

Although we have taken a Bayesian approach (involving formulation and updating of prior distributions), we have assumed that the resulting posterior will be interpreted using a hypothesis testing approach.^12^ One alternative to a hypothesis testing approach would be to focus on the eventual precision of the posterior distribution.^12^, ^57^ Alternatively, sample sizes could be selected to optimise a utility function such as the “Expected Net Benefit” under an economic model.^58^, ^59^ This is the Expected Value of Sample Information (EVSI) approach.^17^, ^28^ The basic approach of averaging across a prior distribution is identical: as noted by Sutton et al,^19^ “expected power” calculations might be considered a “half‐way house” between traditional sample size and EVSI calculations. The need for care in choosing a prior distribution, informed by a meta‐analysis, in the EVSI setting has been made clear.^26^, ^27^, ^28^, ^29^, ^30^ Whatever approach to trial design is used, we emphasize the need for the same level of care.

## “EXPECTED POWER” OF THE NEW TRIAL TO IMPACT UPON THE ESTIMATED RANDOM EFFECTS MEAN

We make the simplifying assumption that *τ* is fixed and known. We denote the target parameter by *θ*.

The current estimate of the random effects mean from the meta‐analysis is

$${\hat{\theta }}_{0}\equiv \frac{\sum \left\{y_{i}/\left({\mathrm{se}}_{i}^{2}+\tau^{2}\right)\right\}}{\sum \left\{1/\left({\mathrm{se}}_{i}^{2}+\tau^{2}\right)\right\}}=V\left({\hat{\theta }}_{0}\right)\sum \frac{y_{i}}{{\mathrm{se}}_{i}^{2}+\tau^{2}},$$

where $V\left({\hat{\theta }}_{0}\right)\equiv \frac{1}{\sum \left\{1/\left({\mathrm{se}}_{i}^{2}+\tau^{2}\right)\right\}}$.

The new trial provides data, *y*_new_ ∣ *δ*∼Normal(*δ*, *σ*^2^/*n*), where *δ*∼Normal(*θ*, *τ*^2^).

If we update the random effects meta‐analysis with this new data, we obtain

$$\begin{array}{l} {\hat{\theta }}_{1}=\frac{\sum \left\{y_{i}/\left({\mathrm{se}}_{i}^{2}+\tau^{2}\right)\right\}+\left\{y_{\mathrm{new}}/\left[\left(\sigma^{2}/n\right)+\tau^{2}\right]\right\}}{\sum \left\{1/\left({\mathrm{se}}_{i}^{2}+\tau^{2}\right)\right\}+\left\{1/\left[\left(\sigma^{2}/n\right)+\tau^{2}\right]\right\}}=V\left({\hat{\theta }}_{1}\right)\left(\frac{{\hat{\theta }}_{0}}{V\left({\hat{\theta }}_{0}\right)}+\frac{y_{\mathrm{new}}}{\left(\sigma^{2}/n\right)+\tau^{2}}\right),\\ V\left({\hat{\theta }}_{1}\right)=\frac{1}{\sum \left\{1/\left({\mathrm{se}}_{i}^{2}+\tau^{2}\right)\right\}+\left\{1/\left[\left(\sigma^{2}/n\right)+\tau^{2}\right]\right\}}=\frac{\left[\left(\sigma^{2}/n\right)+\tau^{2}\right]V\left({\hat{\theta }}_{0}\right)}{V\left({\hat{\theta }}_{0}\right)+\left(\sigma^{2}/n\right)+\tau^{2}}.\; \end{array}$$

Say that we are interested in testing the hypothesis,

*H*_0_ : *θ* > *θ*^*^ versus *H*_1_ : *θ* < *θ*^*^, for some *θ*^*^ ≤ 0.

We calculate the “expected power” of this test, as described by Spiegelhalter et al.^11^ The null hypothesis will be rejected if

$${\hat{\theta }}_{1}+Z_{1-\alpha }\sqrt{V\left({\hat{\theta }}_{1}\right)}<\theta^{*}.$$

That is, we will reject the null hypothesis if

$$y<\frac{\theta^{*}V\left({\hat{\theta }}_{0}\right)+\left(\theta^{*}-{\hat{\theta }}_{0}\right)\left[\left(\sigma^{2}/n\right)+\tau^{2}\right]-Z_{1-\alpha }\sqrt{\left[\left(\sigma^{2}/n\right)+\tau^{2}\right]V\left({\hat{\theta }}_{0}\right)\left[V\left({\hat{\theta }}_{0}\right)+\left(\sigma^{2}/n\right)+\tau^{2}\right]}}{V\left({\hat{\theta }}_{0}\right)}.$$

Now, averaging over the prior $\theta ∼\text{Normal}\left({\hat{\theta }}_{0},V\left({\hat{\theta }}_{0}\right)\right)$,

$$y∼\text{Normal}\left({\hat{\theta }}_{0},\frac{\sigma^{2}}{n}+\tau^{2}+V\left({\hat{\theta }}_{0}\right)\right).$$

Under this distribution, the probability of rejecting the null hypothesis (“expected power”) is

$$1-\Phi \left(\sqrt{\frac{\left(\sigma^{2}/n\right)+\tau^{2}}{V\left({\hat{\theta }}_{0}\right)}}Z_{1-\alpha }+\left(\frac{{\hat{\theta }}_{0}-\theta^{*}}{\sqrt{V\left({\hat{\theta }}_{0}\right)}}\right)\sqrt{1+\frac{\left(\sigma^{2}/n\right)+\tau^{2}}{V\left({\hat{\theta }}_{0}\right)}}\right),$$

or denoting ${\hat{\theta }}_{0}=\theta_{0}$ and $V\left({\hat{\theta }}_{0}\right)=\sigma^{2}/n_{0}$,

$$1-\Phi \left(\sqrt{\frac{\left(\sigma^{2}/n\right)+\tau^{2}}{\left(\sigma^{2}/n_{0}\right)}}Z_{1-\alpha }+\left(\frac{\theta_{0}-\theta^{*}}{\sigma /\sqrt{n_{0}}}\right)\sqrt{1+\frac{\left(\sigma^{2}/n\right)+\tau^{2}}{\left(\sigma^{2}/n_{0}\right)}}\right).$$

(Note also that expression (2) in the paper can be obtained by setting *τ* = 0.)

*When is a Bayesian analysis of the new trial data equivalent?*

Say that we take as a prior distribution $\theta ∼\text{Normal}\left({\hat{\theta }}_{0},V\left({\hat{\theta }}_{0}\right)\right)$.

For equivalence with the analysis/expected power calculation above, we would have to assume that the likelihood of the new trial data was *y*_new_∼Normal(*θ*, (*σ*^2^/*n*) + *τ*^2^), ie, that the new trial estimates the target parameter, *θ*, with some additional random error represented by the variance term *τ*^2^.

Then, the posterior distribution would be

$$\theta |y_{\mathrm{new}}∼\text{Normal}\left(\frac{y_{\mathrm{new}}V\left({\hat{\theta }}_{0}\right)+{\hat{\theta }}_{0}\left[\left(\sigma^{2}/n\right)+\tau^{2}\right]}{V\left({\hat{\theta }}_{0}\right)+\left(\sigma^{2}/n\right)+\tau^{2}},{\left(\frac{1}{V\left({\hat{\theta }}_{0}\right)}+\frac{1}{\left(\sigma^{2}/n\right)+\tau^{2}}\right)}^{-1}\right).\;$$

The mean and variance of this posterior distribution simplify to ${\hat{\theta }}_{1}$ and $V\left({\hat{\theta }}_{1}\right)$ above.
